# Supplementary material for: Anti-SARS-CoV-2 Activity of Polysaccharides Extracted from Halymenia floresii and Solieria chordalis (Rhodophyta)
Source: Mar Drugs. 2023 Jun 6;21(6):348. doi: 10.3390/md21060348 (PMC10305432; doi:10.3390/md21060348)
Supplement: Supplementary file 1 [file marinedrugs-21-00348-s001.zip › marinedrugs-2356633-supplementary.pdf]

Table S1. Biochemical composition (% of dry weight) of Sulfated Polysaccharides fractions 1.1 to 1.6 of the red seaweeds *Halymenia floresii* and *Solieria chordalis*

Fraction	Seaweed species	Extraction-Purification		Neutral sugars	Sulfate groups	Uronic acids	3.6-AG	Protein	Mineral matter
1.1		EAE	Dialysis	24.7 ± 1.6	17.2 ± 1.9	1.9 ± 0.2	1.0 ± 0.02	2.7 ± 0.3	31.1 ± 1.2
1.2	*Halymenia*	HWE	Dialysis	24.9 ± 2.1	18.6 ± 2.3	2.1 ± 0.3*	1.2 ± 0.01	2.9 ± 0.2	33.9 ± 3.2
1.3	*floresii*	EAE	IEX	36.9 ± 0.04	9.4 ± 0.1	3.16 ± 0.05	0.6 ± 0.04	1.18 ± 0.03	/
1.4		HWE	IEX	37.0 ± 0.05	10.1 ± 0.02*	3.13 ± 0.04	0.5 ± 0.02	1.12 ± 0.03	/
1.5	*Solieria*	EAE	IEX	22.8 ± 0.7	15.4 ± 0.02	7.6 ± 0.1	8.4 ± 0.3	7.5 ± 0.1*	/
1.6	*chordalis*	HWE	IEX	23.7 ± 0.5	13.5 ± 0.4*	6.7 ± 0.09*	8.8 ± 0.08	6.2 ± 0.02	/

EAE: Enzyme-Assisted Extraction with protamex; HWE: Hot Water Extraction; IEX: Ion exchange Chromatography (DEAE-Sepharose fast-flow); 3.6-AG: Anhydrogalactose; /: Not determined; Data are means ± SD (n=3). *Significantly different ($p<0.05$) between EAE and HWE by biochemical group (one-way ANOVA).

Table S2. Monosaccharide composition of polysaccharides Fractions 1.1 (Enzyme-Assisted Extraction) and 1.2 (Hot Water Extraction) of *Halymenia floresii*

Fraction	Monosaccharide (% of the total)						Total (µg/mg dw)
	Arabinose	Glucosamine	Galactose	Glucose	Gluc-Ac	NI	
1.1	8.19 ± 0.4	0.17 ± 0.08*	69.9 ± 1.2	10.7 ± 1.2	2.33 ± 0.1	8.61 ± 0.3	139.8 ± 1.9
1.2	9.69 ± 0.6	0.06 ± 0.009*	72.5 ± 2.2	8.87 ± 0.4	2.51 ± 0.2	6.3 ± 1.3	137.9 ± 3.9

Gluc-Ac: Glucuronic acid; NI: Non-identified representing the sum of non-identified monosaccharides. Data are the mean of duplicates (n=2). *Significantly different ($p<0.05$) by column between EAE and HWE for each monosaccharide (one-way non-parametric ANOVA Kruskal-Wallis).
